# Proposing a sequential comparative analysis for assessing multilateral health agency transformation and sustainable capacity: exploring the advantages of institutional theory

**DOI:** 10.1186/1744-8603-10-38

**Published:** 2014-05-20

**Authors:** Eduardo J Gómez

**Affiliations:** 1 King's College London, International Development Institute, Room 7G, Chesham Building, Strand, London WC2R 2LS, UK

## Abstract

**Background:**

This article proposes an approach to comparing and assessing the adaptive capacity of multilateral health agencies in meeting country and individual healthcare needs. Most studies comparing multilateral health agencies have failed to clearly propose a method for conducting agency comparisons.

**Methods:**

This study conducted a qualitative case study methodological approach, such that secondary and primary case study literature was used to conduct case study comparisons of multilateral health agencies.

**Results:**

Through the proposed Sequential Comparative Analysis (SCA), the author found a more effective way to justify the selection of cases, compare and assess organizational transformative capacity, and to learn from agency success in policy sustainability processes.

**Conclusions:**

To more affectively understand and explain why some multilateral health agencies are more capable of adapting to country and individual healthcare needs, SCA provides a methodological approach that may help to better understand why these agencies are so different and what we can learn from successful reform processes. As funding challenges continue to hamper these agencies' adaptive capacity, learning from each other will become increasingly important.

## Background

Reforming multilateral health agencies, such as the World Health Organization (WHO), the Global Fund to Fight HIV/AIDS, Tuberculosis, and Malaria (Global Fund), and UNAIDS for greater effectiveness in meeting healthcare needs is an important area of research. As nations face challenges effectively responding to disease, demands on these and other multilateral agencies have increased. These agencies find themselves in a difficult position, as the ongoing global recession has led to a decline in government and private sector contributions to these agencies at a time when country needs have increased. This problem has motivated scholars to examine the willingness and capacity of multilateral health agencies to overcome these challenges, transform and sustain their policy missions [1,2].

This study claims that better understanding and explaining this process may benefit from a new comparative methodological approach to examining if, why, and how multilateral health agencies not only reform their organizations and policies but also how they sustain reforms over time. Indeed, what seems to be missing in the literature is a clearly defined, systematic methodological approach for comparing, analyzing, and explaining both the reform of multilateral health agencies and the *sustainability* of reforms. Here, I define *sustainability* as the ongoing funding of policy innovations as well as the creation of venues for new policy ideas and learning. However, to better understand why some agencies achieve sustainability and why others do not, one must first analyze and compare the willingness and reform capacity of multilateral health agencies.

In an effort to achieve this, this study introduces a comparative method for analyzing multilateral health agencies. Drawing from the social science institutional theory literature, I propose a *Sequential Comparative Analysis* (*SCA)* to comparing reform and sustainability processes within multilateral health agencies. Through this approach, investigators may use institutional theory, such as path dependency and institutional change theory, to select agencies for comparative analysis. These theoretical frameworks are used to choose agencies that represent theoretical – rather than empirical - issues and concerns. Researchers then compare agencies confronting similar types of theoretical issues in order to discover and explain differences in organizational and policy reform, concluding with a comparison of those agencies that have sustained their reform efforts.

In this study, the cases of the WHO, the World Bank, and UNAIDS were selected because they a) were emblematic of a particular institutional theoretical approach; and b) because they either provided an example of the failure of multilateral health agencies to achieve reform or were a good example of institutional change and sustainability processes. Such an approach is suitable when the goal is to learn more about particular case studies while providing new insights into a topic that has already been investigated [3].

## Methods

This article conducts a qualitative methodological approach to comparative analysis. Empirically, this study relied on various published sources, such as media and peer-reviewed journal articles, books, and reports from multilateral health agencies. In terms of methodology, this article uses an *analytical narratives* perspective [4]. According to this approach, the goal is to select case study examples as illustrations of the potential efficacy of a theoretical approach. The objective is therefore not to randomly select case studies in order to test established theories with the hopes of creating a generalizable claim.

### Sequential comparative analysis and institutional theory

To better understand and explain the complex processes involved in reforming multilateral health agencies, a methodological approach for selecting and comparing agencies is needed. To fill in this lacuna, this study proposes a methodological approach called a *Sequential Comparative Analysis* (SCA) to comparing and analyzing multilateral health agencies. This approach is *sequential* and *comparative* because it compares and explains reforms within multilateral health agencies, over time. And it is *analytical* because it uses institutional theory to guide the selection of those agencies to be compared. Therefore, agencies are not chosen for their similar organizational structure and policies – e.g., agencies falling under the UN system, or philanthropic institutions, such as the Bill & Melinda Gates foundation, but for their illustration of the utility of a particular institutional theory.

**Figure 1:**
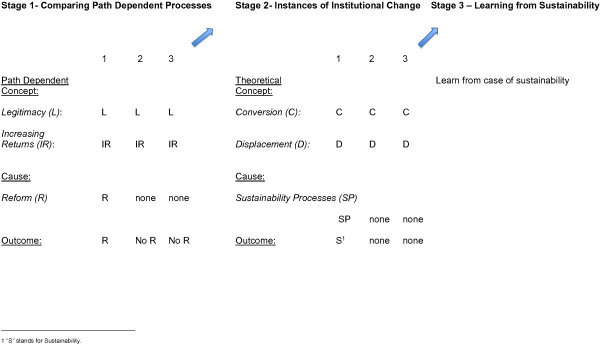
Sequential Comparative Analysis (SCA).

As the first step in this approach, and as Figure 1 illustrates, Stage 1 entails selecting and comparing multilateral health agencies that provide examples of a particular institutional theory, such as path dependency. Path dependency theory is selected because it is a school of thought explaining why institutions often fail to reform for greater effectiveness [5,6]. Path dependency takes a historical approach to explaining why individuals within institutions often fail to engage in more efficient reform processes, even when they are aware of their institutional and/or policy inefficiencies (ibid). Several concepts and causal mechanisms account for these inefficiencies.

For example, institutional *legitimacy* and *learning* creates incentives for individuals not to pursue institutional reforms. Problems of institutional *legitimacy* arise when policy-makers adhere to a particular set of institutions, regardless of their known inefficiencies, mainly because of the institution’s widespread support among trusted peers; this support arises because of the institution’s repeated track record of success, as well as peers’ subjective beliefs that it is the appropriate institution to select [2,5,7]. While alternative and more effective institutions may be present, they are avoided, even without engaging in rational cost-benefit analysis (ibid). Alternatively, the challenge of institutional *learning* occurs when individuals receive a high level of knowledge and training into a particular policy and/or institutional approach [8,9]. Because of their extensive knowledge and training, as well as the passing down of this knowledge from peers, policy-makers feel comfortable and confident in a particular policy/institution; as a result, they believe that it stands above any alternative, more effective approach.

Other path dependency theories focus on resource constraints within institutions, such as *increasing returns* – synonymous with *sunk costs* theory [8,10,11]. The challenge of increasing returns emerges when individuals initially invest an excessive amount of financial and technical resources into an institution, ultimately making it too costly to switch to another more efficient institution notwithstanding the known inefficiencies associated with the existing institution (ibid). When discussing the production of typewriter keyboards, for example, David [11] claimed that because firms invested too much money and technical training into the initial construction of the keyboard, despite the keyboard eventually being perceived as too ergonomically difficult – e.g., its QWERTY letter design, because an excessive amount of resources and training had already been invested in constructing the keyboard, the organizational costs of switching to another more ergonomically efficient keyboard greatly exceeded the benefits [8,10,11].

These path dependency theories may then be used to select multilateral health agencies exhibiting instances of these theorized problems. For example, and as Figure 1 illustrates, the researcher may choose agencies that exhibit the aforementioned *legitimacy* and *increasing returns* constraints. Questions derived from these theories, such as why individuals within agencies repeatedly refrain from adopting new policies, may guide the analyst’s selection of cases. Researchers then search for agencies and empirical case study literature suggesting this repeated problem.

The next step is to compare multilateral health agencies in order to see which of these agencies eventually pursues reform. The goal is to discover the causal mechanisms that led to institutional change – denoted in Figure 1 as “Cause.” Individuals within multilateral agencies may successfully pursue reforms because of their strategies to work with external actors that help legitimize their call for reform [12]. Others may instead appeal to either the media, multilateral agencies, or transnational health movements to delegitimize the agency’s policies (ibid). An eventual outcome arises where multilateral agencies vary in their ability to pursue reform (“R”) or not – denoted as “R” or “No R” in Figure 1.

Next, the researcher transitions to Stage 2 in order to better understand and explain how multilateral health agencies pursued institutional change. In addition to selecting cases, institutional change theory is used to better understand and explain the reform process. Without this theoretical approach, it is harder for researchers to know what they are looking at, i.e., the relevance of particular agency officials, their interaction with other officials and the international community [13].

At its core, institutional change theory examines the conditions under which individuals decide to break away from path dependent processes and pursue reforms. In contrast to path dependency, historical analysis is not as important; rather, the confluence of interactions between endogenous actors and exogenous conditions is (Mahoney and Thelen [12,14]). For example, a process of institutional *conversion* explains how individuals within institutions use changes in the environment, such as international criticisms and pressures, as well as supportive external allies to re-shape and use existing institutions and policy procedures for alternative, more effective policy ends (ibid; [13]). Alternatively, an instance of institutional *displacement* occurs when individuals within institutions work with supporters outside of their institutions and use similar changes in the environment to completely transform their institution’s formal design and policy objectives [12]. And an instance of institutional *de-legitimacy* occurs when individuals within institutions seek to discredit their leaders by repeatedly highlighting their shortcomings and failed policy objectives (ibid).

As I explain shortly, the case of the World Bank and UNAIDS may provide good examples of institutional *conversion* (C) and *displacement* (D) theory. Next, the researcher compares these instances of agency transformation in order to discover key causal differences in these agencies’ ability to engage in policy *sustainability* – denoted in Figure 1 as *Sustainability Processes*. This study considers agency *sustainability* as entailing two factors. First, *sustainability* often requires that agencies ensure that they continuously finance new policy initiatives. And second, *sustainability* requires an agency’s ongoing receptivity to policy ideas and information; obtained from publications, consultation from academics and even social media, this helps to obtain information, learn about healthcare needs and adjust policies appropriately.

SCA’s final Stage 3 entails selecting cases of successful *sustainability* and learning from them. Here, the investigator selects a successful case(s) of agency *sustainability* and strives to discover the causal mechanisms that can help agencies sustain innovative responses to global health challenges.

But why is the SCA method important? This methodological approach is important for several reasons. Chief among them is the realization that, to the author’s knowledge, there are no systemically clear methodological approaches for comparing and analyzing multilateral health agencies. While the works of Gómez [2], Gómez and Atun [15], and Chorev [16] compare several multilateral health agencies, they do not propose a systematic method for comparative analysis.

Second, those studies that do exist choose and compare multilateral health agencies based on their *empirical* problems. That is, health agencies are not selected and guided by institutional theory; instead, they are selected in order to provide insights into the empirical organizational, financial, human resource, and policy problems hampering health agency performance. For example, the works of Peabody [17], Glassman and Savedoff [18], and Chorev [16] address these challenges within the WHO and Global Fund and how they have constrained the governing board’s ability to pursue reforms. Alternatively, case studies are selected in order to explain an agency’s success in overcoming these empirical challenges [19].

Additionally, efforts exist to compare and assess the performance of multilateral agencies, such as the Multilateral Organizational Performance Assessment Network (MOPAN). MOPAN is comprised of a network of 17 donor countries working together to analyze the effectiveness of the multilateral agencies that they fund through the collection of survey data, documents discussing agency performance in achieving objectives, as well as information obtained through staff interviews [20]. Known as the “Common Approach” methodology, through these efforts MOPAN provides a platform for agencies to exchange information and to learn from each other (ibid). Nevertheless, MOPAN’s analysis is not comparative, such that it analyzes case studies on an individual basis [20].

Finally, studies seem to analyze multilateral health agencies within a restricted period of time (Peabody [17-20]). Scant attention is paid to analyzing reform processes over a long period of time; nevertheless, as Pierson [21] maintains, this approach is needed in order to more accurately describe and explain institutional change processes and policy outcomes. SCA achieves this process through its overtime sequential analytical approach.

### Analyzing SCA’s theoretical approach in light of institutional theory

In the social sciences, a myriad of institutional theories exist accounting for the performance of institutions. Addressing this literature and comparing it with SCA’s proposed path dependency and institutional change theories helps to justify why path dependency and institutional change theory was chosen and why these two schools of thought appear to be more advantageous in accounting for similar institutional challenges and change processes within multilateral health agencies.

Several theories have focused on the reasons why institutions often fail to pursue reforms for greater efficiency, eventually leading to a path dependent process; some claim that the challenge of institutional *coordination effects* generates this undesirable outcome [8,22-24]. According to Arthur [25], coordination effects emerge when institutions, such as production industries, benefit from adopting and maintaining a particular technology that other industries are using. Doing this, as well as adopting the rules and regulations associated with the technology, helps to lower the transaction costs of monitoring other industries’ actions, in effect, increasing the predictability of their strategies; but this also facilitates policy planning, while leveling competition and assuring profits. Arthur [25] claims that these “positive network externalities” increase as more institutions join the network and adopt the same technology, as well as the set of rules that go along with being a group member. Because of these benefits, industries avoid pursuing any other form of potentially advantageous technologies because of the potential costs of leaving the coordinating group and the start up costs involved in joining another industrial network (ibid).

*Policy feedback* processes have also generated few incentives to pursue policy and institutional change. According to Skocpol [26] and Pierson [21], a feedback process occurs when the creation of policies and/or institutions leads to the rise of political and civil societal coalitions that, in turn, sustain and safeguard these policies and/or institutions [21,26]. Politicians benefit from safeguarding social welfare benefits, for example, while civil society benefits from continuously receiving social services (ibid). Eventually, these reinforcing political and civil societal coalitions succeed in safeguarding their policy and institutional preferences, even when others perceive them as inefficient, costly, and in need of reform (ibid).

Finally, theorists have offered a *cultural institutional* approach to explaining why actors fail to reform institutions for greater effectiveness. Greif [27] maintains that a long history of cultural (religious) beliefs among particular economic trade groups shapes the emergence of trade institutions, i.e., shared expectations and coordination between merchants and traders, which safeguard their institutional designs at the expense of crowding out more effective alternatives. Grief [27] claims that cultural religious groups will often prioritize their belief systems, norms and expectations over the adoption of other policy ideas and institutions that are proven to be more effective and lucrative. Similarly, others have shown how conservative cultural beliefs within government institutions shapes the rise of enduring condemnatory, inefficient laws and welfare programs [28], while informal cultures of corruption have often hampered the reform of ineffective legal institutions [29].

When compared to each other, however, it seems that SCA’s proposed path dependency theories of *legitimacy*, *learning*, and *increasing returns* are more effective than *coordination effects*, *policy-feedback*, and *cultural institutional* approaches to explaining the challenges of reforming multilateral heath agencies. First, institutional *coordination effects* may not be very helpful because multilateral health agencies often do not adopt each other’s institutional designs and policy ideas; instead, their policies are more often influenced by the agency’s historical institutional formation processes, policy preferences and experiences [15]. In light of the importance of these historical factors (ibid), *coordination effects* may be further limiting because this approach cannot tell us how preexisting multilateral health policies, policy ideas, and experiences shape policy-makers’ views of these policies, their ongoing success, popularity, and, therefore, legitimacy.

In contrast, theories of institutional *legitimacy* and *learning* appear to be better positioned for taking into consideration the importance of historical policy precedents within multilateral health agencies and how these precedents create incentives for decision-makers to refrain from pursuing institutional change. Furthermore, when compared to *coordination effects*, theories of *legitimacy, learning,* and *increasing returns* are more applicable to analyzing multilateral health agencies mainly because these frameworks discuss the interests, behaviors, and consequences of policy-making elites rather than focusing on coordinating relationships between multilateral agencies.

*Policy-feedback* processes also provide a limited approach to analyzing multilateral health agencies. This is mainly because feedback processes focus on analyzing institutional decision-makers *as well as* the civil societal actors that they collude with, as the feedback process depends on the success of their coalitional partnership. Nevertheless, policy-making within multilateral health agencies has often been driven by agency elites, such as governing board members and presidents, not civil society [15]. Perhaps with the exception of institutions such as the Global Fund to Fight HIV/AIDS, Tuberculosis, and Malaria, policy-making and policy sustainability processes never depend on the support of civil societal actors (ibid).

Finally, a *cultural institutional* approach may provide insight into the challenges that multilateral health agencies face when considering reforms. Indeed, Peabody [17] has argued that the WHO’s preexisting organizational culture of responding to diseases has continued to shape the WHO’s epidemiological surveillance strategies, leading WHO officials to avoid the pursuit of more effective surveillance measures. Nevertheless, the challenge with a cultural institutional approach is that it fails to provide clear causal mechanisms into precisely *how*, as well as *which* types of cultural beliefs (religious or non-religious, e.g., organizational) shape policy-makers’ perceptions, interests, and reform strategies. Are certain cultural beliefs passed on to other multilateral agency officials? If so, how and which ones? And which types of cultural beliefs affect individuals’ views? Is it beliefs in preexisting policy experiences, ideas, or routine (seemingly ritualized) managerial practices?

In contrast, theories of *legitimacy* and *learning* not only provide clear causal mechanisms illustrating how prior policy ideas and experiences influence agency officials’ policy-making decisions, but these theories also clarify that it is preexisting policy beliefs, experiences, popularity, and peer support, rather than routine managerial practices, that motivates individuals to refrain from pursuing institutional change.

In addition to explaining institutional stasis, recently scholars have also explained the conditions under which institutions eventually change for greater effectiveness. Deeg [30,31], for example, has emphasized the importance of *international pressures* in fostering institutional change. For instance, domestic economic institutions, such as banks, may at times confront international pressures to comport with new kinds of financial transactions, accounting standards, and technologies, in turn motivating bankers to reform their outdated banking procedures in order to comply with international expectations and standards (ibid). Others have argued that international pressures also motivate ministries of health to reform their policies and bureaucratic structure in order to bolster their international reputation for having effective public health programs [32,33].

However, institutional change does not have to be the product of international pressures. Institutions may also gradually evolve on their own [12,14,34]. Some argue that domestic political interests and strategies within institutions are more important. According to several scholars [12,34-37], institutional *layering* theory, for example, emphasizes this domestic approach, positing that when reformers within institutions face considerable political resistance to institutional change, they will instead add new institutions, such as legislative committees and/or agency subdivisions, on top of existing institutions in order to avoid ongoing political conflict, circumvent ineffective institutions, and achieve their policy objectives.

Finally, others have argued that civil societal interests, pressures, and mobilization strategies are important for incentivizing governments to pursue institutional change [7,38,39]. Civic movements succeed in using formal and/or informal institutions to not only funnel their interests but also to establish coalitions with those reform actors seeking institutional change (ibid). According to Weir [38], institutional change can only take place when policy-makers are socially “embedded” and influenced by civil society. Here, civic organizations and/or interest groups take the lead in establishing reform coalitions with supportive politicians and bureaucrats while using existing institutional channels, such as legislative committee hearings, to magnify their voice and influence (ibid; [40]).

But how does SCA’s recommended institutional change theories of *conversion* and *displacement* compare to these other institutional change theories when accounting for reform within multilateral health agencies? And which theoretical frameworks appear to be more helpful for understanding this process?

With respect to *international pressures*, while this approach may provide insight into the reasons why multilateral health agencies pursue reform, it does not provide a precise analytical explanation for *how* reform actors within institutions use international pressures to assist them in their cause. Do they ask other multilateral health agencies to lobby the reform actor’s governing board on their behalf, or perhaps NGOs and the media? In contrast, the advantage of institutional *conversion* theory is its ability to precisely explain not only which reform actors within multilateral health agencies strive to take advantage of international pressures (e.g., marginalized agency officials), but more importantly, how they strategically use these pressures in order to increase their legitimacy and influence when pursuing reform [12]; as I discuss later with the case of the World Bank, this can be achieved by agency officials not only alluding to international pressures and criticisms but also through their efforts to establish coalitional partnerships with other supportive multilateral agencies.

Alternatively, institutional *layering* could provide insight into how reform actors within multilateral health agencies circumvent internal resistance to reforming their bureaucracy through the creation of additional committees building on top of preexisting committees. Nevertheless, the application of this theory would leave us wondering how and why these reformers were empowered to create additional committees; what precisely were their sources of strength and influence and where did these sources come from? In contrast, institutional *conversion* and *displacement* theory seems better positioned to answer these questions: this is because these theories can help to illustrate how reformers strategically use changing international environments and supportive allies in other multilateral health agencies to discredit inefficient institutions, legitimize and empower their reform efforts. *Conversion* and *displacement* therefore provide a fuller explanation for the originating sources of reformers’ strength and influence.

Finally, with respect to civil societal approaches to institutional change, while this theoretical approach may help to explain reform processes within democratic political institutions, it is doubtful that civil society has had just as much influence within multilateral health agencies. For instance, while the World Bank and the Asian Development Bank (ADB) has created several committees that allow civil societal actors to express their dissatisfaction with health and other social welfare policies while potentially striving to build partnerships with World Bank reformers [41,42], scholars find that the World Bank and ADB’s governing boards and executive directors have not been fully committed to incorporating the views of their representative civil societal institutions [41,43]. Perhaps the one exception would be the Global Fund, where civil societal representatives – i.e., victims of disease and the private sector – are present on the governing board.

Thus, because the influence of civil societal pressures on multilateral health agencies is questionable, institutional *conversion* and *displacement’s* focus on reform actors within multilateral health agencies, as well as their strategic usage of external conditions and allies in other multilateral agencies, appears to be more applicable and advantageous for understanding and explaining the transformation of these institutions.

### Empirical analysis and sequential comparative analysis (SCA)

#### Stage 1 – Comparing path dependent processes

As Gómez and Atun [15] explain, multilateral health agencies falling under the UN’s governance structure exhibit similar types of originating political coalitions, interests, and designs in governing board accountability and policy incentives. Yet, these UN agencies, such as the WHO, UNAIDS, and the World Bank, can also be compared to each other because of their similar path dependent challenges, such as institutional *legitimacy* and *increasing returns*. Historically, these path dependent constraints have hampered efforts to reform these agencies for greater effectiveness in providing technical and financial assistance while meeting country needs (ibid; [2]).

The WHO provides a good illustration of how institutional *legitimacy* and *increasing returns* poses obstacles to achieving these outcomes. Peabody [17] states that the WHO governing board and staff initially adopted the YAWS^a^ approach to epidemiological evaluation, technical assistance, and organizational management as the most effective response to disease outbreaks. During the 1970s, all WHO staff were trained in this approach. In line with *increasing returns* theory, a high level of financial and technical investment went into YAWS (Peabody, [17]). These initial investments underscored the high level of confidence and belief in YAWS. Consequently, Peabody [17] claims that an organizational culture arose whereby YAWS was perceived by WHO staff as the most legitimate and effective course of action to take in response to disease outbreaks (ibid). Gómez [2] and Peabody [17] also claim that over time, WHO staff learned and passed on this approach to other staff members.

Nevertheless, when new types of diseases emerged, the *legitimacy* and *increasing returns* associated with YAWS posed challenges to agency adaptability and response to country needs. For instance, Peabody [17] claims that when the HIV/AIDS epidemic emerged, WHO leaders, such as Director General (DG) Hiroshi Nakajima, proposed an alternative approach to studying HIV/AIDS, which included examining preventative measures in the form of condom use and education. WHO staff nevertheless resisted Nakajima’s suggestions because they were not seen as legitimate and effective [2,17]. Moreover, it seems that the high level of initial investment into YAWS contributed to this sense of resistance, as directors and staff found it too difficult to shift to a new disease surveillance approach [2].

Years later, when DG Gro Bruntland emerged to strengthen organizational efficiency [44], as well as partnerships with the private sector and WHO country office capacity, Horton [45] and McCarthy [46] claim that WHO staff resisted her reforms because they were perceived as illegitimate and ineffective. While Bruntland succeed in establishing these partnerships, she did not succeed in strengthening organizational efficiency [47]. The same dilemma occurred under DG Lee Jong-Wook’s efforts to introduce reforms such as job rotation, transparency, and the allocation of resources to WHO regional offices [2,48]. Since assuming office, similar *legitimacy* challenges have challenged DG Margaret Chan’s efforts to strengthen the WHO’s organizational capacity and responsiveness to country needs [2]. WHO staff have resisted Chan’s ongoing hierarchical approach to decision-making and policy implementation (ibid; [49]). Much of this resistance stems from the staff’s belief in their own approach to organizational management and structure, which places an emphasis on what they know best: participatory decision-making and agency autonomy, hallmarks of the YAWS approach (Peabody, [2,17]).

The World Bank also faced similar path dependent *legitimacy* problems shortly after its creation in 1944. Although the Bank was initially designed as an institution providing loans for economic reconstruction in war-torn Europe [50,51], by the 1950s pressures were increasing for assistance to help nations combat poverty [50]. Board members, as well as World Bank economists, started claiming that investing in health, education, and infrastructure facilitated timely economic development (ibid).

Nevertheless, the *legitimacy* of prior lending procedures complicated the Governing Board and staff’s ability to transform the World Bank’s lending policies. Lending for economic restructuring, as well the financial conditionalities imposed for receiving assistance, were policy traditions passed down from one Governing Board and Executive Director to the next [52]. Board members and Bank staff believed that these policies were an effective and legitimate course of action to take (ibid). Therefore, when the idea of introducing anti-poverty programs emerged, first introduced under Bank President Robert McNamara (1968-1981) [53], board and staff members vehemently resisted the notion, seeing these types of policies as illegitimate and financially irresponsible (ibid). This difference between increased international demands for Bank policies in poverty alleviation, President McNamara’s support for these policies, versus Bank staff preferences to resist such policies confirms other scholars’ view that the Bank has often behaved in a hypocritical manner [54].

UNAIDS also faced similar institutional *legitimacy* challenges. Created in 1996 in response to the UN’s inability to effectively coordinate a response to AIDS [55], UNAIDS was created in order to increase UN inter-agency coordination, encourage other UN agencies to contribute their expertise and experience, increase and facilitate the sharing of resources, technical expertise, and policy-making capacity [19,56,57]. 10 UN agencies, also known as “cosponsoring organizations,” fell under the leadership of the UNAIDS Secretariat, which is primarily responsible for providing technical and administrative assistance to UN cosponsors and their AIDS programs [19]. UNAIDS is also governed by a Programme Coordinating Board, comprised of 22 Member States, the UN cosponsoring organizations, NGOs, activists, and people living with HIV/AIDS (ibid).

Nay [19] nevertheless claims that the office of the UNAIDS Secretariat was initially limited in its ability to achieve its responsibilities, coordinate and find consensus for policy reform. In addition to having an insufficient amount of financial and technical resources to adequately perform its duties, the Secretariat confronted a crisis of institutional *legitimacy* within UNAIDS (UNAIDS, [58]). This limited the Secretariat’s ability to pursue reforms; this was mainly attributed to the fact that other co-sponsoring UN agency directors believed that the most legitimate and effective way to coordinate and reach a consensus over reform was to engage in open discussions and consensus-building between other UN agency cosponsors, not contributing UN Member States, which was initially proposed by the UN ECOSOC (Economic and Social Council) (UNAIDS, [19,58]). These conflicting views contributed to a high level of internal competition and contestation within UNAIDS (ibid). When the Secretariat tried to increase his influence in building a consensus and facilitating coordination between participating UN agency cosponsors and the Member States, UN agency cosponsors viewed this endeavor as an illegitimate, ineffective move (ibid). UN agency cosponsors displayed their views and discontent by essentially ignoring the Secretariat’ efforts (ibid).

While the WHO, the World Bank and UNAIDS all experienced initial path dependency challenges, some of these agencies eventually succeeded in pursuing reform. As Gómez [2] explains, the World Bank and UNAIDS eventually proved capable of reforming their bureaucratic procedures and policies, in turn strengthening their response to international and country needs. But how did the World Bank and UNAIDS achieve this? Addressing this question takes us to Stage 2 of SCA.

#### Stage 2 – Instances of institutional change

Stage 2 of SCA requires that the researcher use institutional theory, such as institutional change theory, to select cases and to explain how and why those multilateral health agencies examined during Stage 1 eventually transformed. At this stage, the researcher may choose from the aforementioned menu of institutional change theories, such as *conversion, displacement,* and *de-legitimacy*, to more accurately explain why and how these agencies transformed. Using institutional change theory and its causal mechanisms helps to organize and make sense of the complex interplay between exogenous and endogenous conditions, agency interests, and reform strategies [2,13].

When it came to the World Bank, institutional *conversion* helps to explain why the Bank eventually decided to break away from its aforementioned path dependent tendencies and decided to change its policy mandate from focusing on lending for economic restructuring to funding health and social welfare policies. According to institutional *conversion* theory, previously marginalized individuals within an institution seek to re-use existing institutional rules and policy procedures for new policy ends. When new exogenous criticisms and pressures emerge, these marginalized individuals strategically use these external pressures to discredit existing policies and their supporters [12,14]. At the same time, these individuals seek influential allies, who are often located outside of their institution, in order to strengthen their legitimacy and influence [14].

This is precisely what occurred at the World Bank during the 1960s. As Ruger [53] explains, by the early-1960s the Bank was exposed to intensive international criticisms and pressures to transform its policy focus from strictly providing loans for economic reconstruction to providing loans for poverty, education, and health. Though ignored for several years, members of the World Bank’s IDA (*International Development Association*), an arm of the World Bank providing financial assistance to low income countries [52], emerged to strategically use these international criticisms in order to underscore the Bank’s inequitable policies, as well as the fact that they were not helping nations emerge out of poverty [2]. IDA staff members believed that financing health and education was the best way to eradicate poverty [59].

Furthermore, Gómez [2] writes that in tandem with these exogenous pressures, IDA staff sought to strengthen its ties with supportive leadership staff at the WHO and UNESCO [59]. By establishing a strong partnership with these agencies, IDA staff was able to legitimize their efforts, their proposed policy ideas and influence within the Bank (ibid; [52]). By the early-1960s, these efforts paid off: the IDA was able to succeed in convincing the Bank Board of Executive Directors that it had to transform its policy priorities from strictly funding economic reconstruction to also funding health, education, and other poverty alleviation programs [2,52].

UNAIDS also joined the World Bank in eventually breaking away from the aforementioned path dependent inefficiencies of institutional *legitimacy*. Theoretical processes of institutional *displacement* help to explain how this occurred. *Displacement* processes arise when previously marginalized individuals within institutions strategically use a change in the external environment, such as international pressures or crisis situations, to delegitimize existing institutional procedures and policies and replace them within new ones, establishing new policy goals and aspirations [12]. These sudden shifts in the environment in turn empower those individuals seeking institutional change (ibid).

Nay’s [19] discussion of UNAIDS’ transformation since 2005 provides a good example of *displacement* processes. As mentioned earlier, during the 1990s the UNAIDS Secretariat was marginalized, seen as illegitimate in the eyes of co-sponsors. Yet, Nay [19] claims that after the rise of international pressures for a reform of the UNAIDS structure and procedures, the agency began to change. Specifically, Nay [19] states that: “International institutions tend to be path dependent, and only external inducements may have encouraged them to opt for change.” These international pressures emerged from international donors, NGOs and governments claiming that UNAIDS was incapable of adequately responding to country needs, for failing to establish strong partnerships between co-sponsors, insufficient human resource capacity, discrepancies between priorities and objectives, mistrust between managerial teams, as well as a general lack of funding and transparency [19,2]. UN Secretary General Kofi Anan also contributed to this censure, arguing for the need for administrative reform, while organizing international conferences advocating increased harmonization and coordination between UN agencies [19].

Furthermore, Gómez [2] and Nay [19] claim that these international pressures empowered the previously marginalized UNAIDS Secretariat. By strategically appealing to these external criticisms, the Secretariat was able to justify its need to essentially completely revamp the entire UNAIDS structure, as well as reforms that went well beyond the Secretariat’s initial attempt to increase its leadership and coordinating role [2]. By 2005, the Secretariat succeeded in transforming the entire way UNAIDS was governed [60]. New policies were also introduced focusing on performance-based managerial and financial instruments [19,60]; increasing inter-agency coordination through the creation of new steering committees [19,60]; a clear division of labor and responsibilities for policy implementation [19]; and new efforts to make funding and policy decisions transparent.

Thus by the mid-2000s, the World Bank and UNAIDS successfully broke away from their path dependent inefficiencies and pursued institutional change. But the key question to ask is if and how these agencies were capable of *sustaining* their new initiatives and if they differed in the manner in which they achieved this? Addressing this question requires that we compare the World Bank to UNAIDS in order to see which of these agencies engaged in *sustainability* processes, which is denoted as (SP) in Stage 2 of SCA, in Figure 1.

### Sustainability processes

Let us first consider the World Bank. Since the late-1990s, the Bank has sustained its efforts to play an important role in global health through increased funding and program expansion, aided by a substantial rise in Bank Members States’ capital commitments [61]. During this period the Bank also became the largest multilateral financier for health with a portfolio of 154 active and 94 completed projects [62,63]. Governing board members soon realized, however, that they had not properly anticipated the increased demand of country funding for diseases, such as HIV/AIDS (ibid), while also realizing that greater investments in healthcare were needed [64]. In an effort to sustain the Bank’s HIV/AIDS policies, analysts note that “the Bank responded flexibly to the demand for such lending, among other things, with (a) a strategy for intensifying action against HIV/AIDS in Africa in July 1999, (b) the $1 billion Multi-country AIDS Program (MAP) in September 2000 …” (ibid: p. 74). Initiated in 2000, MAP provided a 15 year funding commitment, submitted in 3 phases, with the first considered to be a series of emergency loans, followed by lending for health systems, prevention, community participation and accountability, as well as HIV/AIDS treatment (ibid; [65]). To sustain and strengthen MAP, in 2002 the Bank approved another $500 million in grant funding, which extended MAP financing in 29 countries throughout Africa [66]. Furthermore, in order to further ensure MAP’s success, in 2000 the Bank funded the MAP “Horizontal Adaptable Program Loan” (APL), which incorporates the involvement of civil societal organizations (CSOs), the private sector, ministries outside of the ministries of health, and trans-boundary populations such as refugees [66]. By 2006, in response to continued country needs, the Bank further sustained its commitment to HIV/AIDS by creating the *AIDS Strategy & Action Plan* (ASAP). ASAP is a jointly proposed initiative with UNAIDS that helps nations achieve full country ownership for their HIV/AIDS programs (ibid).

In 2007, as a further sign of the Bank’s sustained commitment to combating HIV/AIDS, it unleashed its *Agenda for Action* (AFA) [66]. With this endeavor the Bank stressed that the “principle goal of the AFA is to reaffirm the Bank’s promise to devote its resources to help halt and begin to reverse the spread of HIV/AIDS” (ibid: p. 6). Through AFA, the Bank is committed to providing $250 million dollars per year for HIV/AIDS initiatives; and to establish an HIV/AIDS grant incentive fund of $5 million dollars annually to promote capacity building (ibid). The overall goal of AFA is to build on ASAP and its emphasis on helping countries develop long-term, sustainable responses to HIV/AIDS; to strengthen the implementation of policy; to enhance Monitoring and Evaluation (M&E); and to strengthen health systems and coordination with donors (ibid).

And finally, realizing that the Bank was no longer the main provider of funding for HIV/AIDS, considering the arrival of funders such as the Global Fund, GAVI, and the Bill & Melinda Gates Foundation, the Bank refocused its policy efforts to emphasize its historic comparative advantage: i.e., health systems strengthening, health financing, and governance (ibid). In fact some claim that in order to sustain and deepen its presence in global AIDS policy, the Bank took on new policy roles, such as transforming from dominant financier to a development partner and complementary funder, which analysts claim required a “larger and more complex role” (ibid: p. 35).

Sustaining the World Bank’s work on global health through the creation of new programs and funding has also benefited from the Bank’s commitment to continuously incorporate external policy ideas. Since the late-1960s, beginning with the Bank’s shift to anti-poverty alleviation strategies, the Governing Board and staff have invited scholars and policy-makers to provide new ideas on how to scale up the Bank’s work in this area [53]. Global health in particular has benefited from the penetration of new policy ideas, reflecting a continued increase in country requests for learning and assistance [53,67]. Since the late-1980s, the Bank has organized several conferences, at times co-sponsored with the WHO, bringing together health policy experts from academia to provide new insights into strengthening health systems and health governance [53,68]. Through the Bank’s *Development Research Group* (DECRG) and the *Social Development Department* (SDV), Bank staff have also periodically invited academics to give presentations on governance and capacity building in response to AIDS [69]. Moreover, from 1997 to 2008, the Bank has funded the provision of over 300 short courses on health systems strengthening through the *World Bank Institute’s Flagship Program on Health Sector Reform and Sustainable Financing*[67]. In recent years, the Bank has also increased its collaboration with the private sector to learn about new innovations in health system strengthening and, more recently, health information technology and management [70]. The Bank has also provided a venue for publishing and disseminating the views of academic scholars and policy-makers on a wide range of health issues [53]. Ruger [53] indeed claims that over the years, the Bank has produced “210 country-specific HNP sector studies and staff appraisal reports and hundreds of country strategy documents on HNP topics.”

When compared to the World Bank, UNAIDS has also been committed to sustaining its commitment to HIV/AIDS by funding new program initiatives. Given the inability of the Global Fund and bi-lateral contributions (especially from the US) to maintain funding commitments, which is mainly due to the ongoing global economic recession [71], UNAIDS has called for a sustainable increase in funding arrangements to make sure that HIV and AIDS victims continue to receive the prevention and treatment services that they need [72]. UNAIDS has not only asked its contributing UN Members States for more funding for its programs [73], but it has also called on new financial innovations and means to raise revenue: “We need new financial modalities and sources of funding such as the financial transaction tax to maintain the momentum of the AIDS response,” argued Mr. Michael Sidibé, UNAIDS Executive Director [72].

In response to this challenging economic context, UNAIDS’ Programme Coordinating Board and Secretariat has been committed to sustaining and increasing their financial commitments to various program initiatives. In response to country needs for greater technical assistance, in 2005, for example, UNAIDS created the Technical Support Facilities (TSF) [74], which are located in approximately 80 countries in Africa and Asia [74]. Through the assistance of country officials and the proactive participation of NGOs, TSFs provides technical assistance for a variety of policies, as well as assistance in grant applications, in order to establish institutional capacity for responding to AIDS [74]. In 2006, and as mentioned earlier, UNAIDS worked with the World Bank to create ASAP, while in 2009, several new prevention initiatives were implemented, such as the *Action Framework for Universal Access for Men who have Sex with Men and Transgender People*[75] and the *Action Framework Addressing Woman, Girls, Gender Equality and HIV*[76]. And in order to further ensure that these Action Frameworks are implemented, in 2010 the *Operational Plan* (2010-14) was created. Managed by UNAIDS staff as well as woman in civil society, while meeting twice a year, the *Operational Plan* further deepens UNAIDS’ commitment to responding to the needs of woman and children by striving to implement three areas of the aforementioned *Action Framework Addressing Woman*: 1) strengthening strategic guidance and support to national governments to “know their epidemic and response,” so as to more effectively meet the needs of woman and girls; 2) help countries in order to make sure that national HIV/AIDS programs, plans, and M&E frameworks address the needs and rights of woman and girls; and 3) advocacy, capacity strengthening and mobilization of resources for the needs of woman and girls in the context of HIV [76].

And in response to UNAIDS’ realization that more work needs to be done with respect to women’s human rights and protection from HIV, in 2011 it was proposed that a UNAIDS Women’s agency be created (ibid). Although no further details have been released to date, this is an innovative and much needed response, further highlighting UNAIDS’ innovative ideas and sustainability of its financial and policy commitments.

Finally, in response to UNAIDS’ Second Independent Evaluation (SIE), which began in 2007 in order to conduct a thorough analysis of the agency’s success, limitations, and suggestions for improvement, the *Getting to Zero* initiative was launched in 2012 [77]. This initiative follows through with the SIE’s recommendation, released in December 2009, that UNIADS exhibit not only stronger leadership but that its programs also become more focused, strategic, flexible, and responsive (ibid). Realizing this need, *Getting to Zero* establishes new goals and processes to achieve zero HIV infections by 2015; to get zero AIDS related deaths by 2015; and to achieve zero discrimination by that date as well (ibid). Following SIE’s 2009 recommendation, then, *Getting to Zero* has a specific focus and goal, while outlining several joint initiatives that can help achieve them.

Finally, UNAIDS has remained open and committed to obtaining feedback from external evaluators, researchers, and civil society on how to improve its policies. In addition to inviting academics to co-author reports on a variety of issues, such as the *UNAIDS Reference Groups* working papers and a myriad of Annual Meeting Reports, UNAIDS has also recently engaged in innovative on-line strategies to obtain information and learn. In 2011, for example, the *CrowdOutAIds.org* initiative was created in order to reach out to the youth about their HIV/AIDS status [78]. Through this on-line interactive service, youth from around the world can communicate with UNAIDS staff about their experiences, provide information and suggestions for how to improve UNAIDS work with them (ibid). To the author’s knowledge, this is the only multilateral health agency that has employed these social media tools to obtain new information and ideas. Therefore, while UNAIDS joins the World Bank in striving to collaborate with external policy experts and academics, it is different from the Bank in that it is exploring alternative ways to learn and adapt to country needs.

In sum, in recent years the World Bank and UNAIDS have been able to successfully engage in *sustainability* processes*.* Yet, there are some differences with respect to how these agencies have achieved this outcome. While both agencies have been committed to increasing their financial commitments to new programs, they have differed in terms of their approach to human resource reform and innovations in acquiring external policy ideas.

#### Stage 3 – Learning from sustainability

But why do these findings matter? Answering this question takes us to Stage 3 of SCA. At this point the goal is to learn from successful instances of multilateral agency sustainability and to provide lessons for other agencies striving to sustain their reform efforts. For instance, the cases of the World Bank and UNAIDS revealed that successful agency sustainability requires that multilateral agencies have the ongoing willingness, commitment and capacity to continuously finance new policy initiatives and respond to country needs. The World Bank and UNAIDS periodically created new policies and changed their policy roles in response to these needs as well as independent evaluations. Nevertheless, this is an endeavor that other multilateral agencies, such as the WHO and the Global Fund, have not been able to achieve ([1,2]; Gómez and Atun, [15]). WHO and Global Fund leaders may therefore wish to learn from the World Bank and UNAIDS’ sustainability strategies.

Second, the cases of the World Bank and UNAIDS suggests that agencies striving for sustainability requires that agency leaders consistently incorporate new policy ideas from academia and/or from civil society. Merely conducting independent evaluations within agencies is inadequate. Rather, agencies should continue to create venues and pro new ones in order to obtain different types of external ideas and policy strategies. The Global Fund, for example, could learn from this approach. When compared to the World Bank and UNAIDS, the Global Fund has not been open to establishing venues for external researchers to provide new ideas about policy and governance issues. Moreover, the Global Fund does not have a formal research department, where it can learn and disseminate information about innovations in health governance and financing. Perhaps it is time that the Global Fund learn from the World Bank and UNAIDS in exploring how policy insights from external researchers can lead to more effective policy interventions.

## Discussion

As multilateral health agencies confront challenging global economic contexts and difficulties in reforming their bureaucracy and policies to meet country needs, scholars and practitioners should strive to compare and analyze why some agencies have overcome these challenges while others have not. This study has argued that we can provide insight into these questions by using social science institutional theories, such as path dependency and institutional change theory, to conduct a *Sequential Comparative Analysis* (SCA) of multilateral health agencies. Path dependency theory can be used to guide the selection of cases that have repeatedly proven incapable of pursuing reforms, as we saw with the WHO, as well as the early years of the World Bank and UNAIDS, while institutional change theory could subsequently be used to select, compare, and explain cases that eventually pursued reform, as we saw with the World Bank and UNAIDS.

SCA may also help to justify why particular agencies are selected and compared. It is often the case that practitioners and scholars simply choose multilateral health agencies for comparison because they exhibit similar political origins, such as agencies emerging from the UN system (Gómez and Atun, [15]); because they share similar types of policy interests and missions; or because they share similar types of governance arrangements, e.g., WHO and UNAIDS versus the Bill & Melinda Gates Foundation and the George Soros foundation. Also, researchers have typically chosen and compared agencies because of their similar *empirical* challenges: e.g., leadership and governance challenges; funding problems; inadequate policy designs and outcomes [15-18]. In contrast, SCA suggests that cases should be selected based on their similar *theoretical* issues and challenges: e.g., instances of the path dependent mechanisms of agency stasis (*learning* and *legitimacy*), as well as the mechanisms of institutional change (*conversion* and *displacement*). Through this comparative approach, we may also discover challenges that affect all types of multilateral health agencies, regardless of their originating structures, policy roles, and missions.

The application of path dependency and institutional change theory also leads to a *sequential* analytical approach to understanding the transformative capacity of multilateral health agencies. That is, an application of both theoretical schools of thought forces researchers to move from an analysis of why agencies do not pursue reform to how and why they eventually do. This, in turn, requires an overtime sequential analysis that better captures and explains the transformative nature of multilateral health agencies. SCA therefore forces scholars to move away from more typical multilateral agency comparisons in finance, human resources, and policy reform at particular moments in time ([16-19]; Nay). This study therefore agrees with Pierson’s [21] notion that only after we have examined independent variables and causal mechanisms over a long period of time can we truly capture and explain the transformative potential of institutions.

Nevertheless, there are potential limitations to SCA’s methodological approach. For example, this study’s proposed definition of agency *sustainability* may be missing several elements that contribute to this process. Perhaps political support and pressures from influential governments; peer pressure from other agencies sustaining their reforms; and bold and creative agency leadership may also explain why agencies engage in sustainability processes. Future researchers will need to expand the number of multilateral agencies assessed and compared to provide more insight into this matter. Relatedly, more case studies will need to be investigated in order to see if the SCA approach is helpful. This article only examined 3 multilateral health agencies; this was done because the goal was to explore and illustrate the potential effectiveness of SCA rather than to confirm SCA’s generalizable application and utility.

But it is also important to note that there are several limitations to the path dependency and institutional change theories used to conduct the SCA comparative method. While earlier in this article I underscored these theories’ advantages when compared to other similar institutional theories, this is not to say that my proposed path dependency and institutional change theoretical frameworks can and should explain *all* types of path dependent and institutional change processes within multilateral health agencies.

For instance, with respect to institutional *learning* and *legitimacy*, these theories may not be very effective when striving to explain institutional stasis and inefficiency within more recently established multilateral health agencies, such as the Global Fund, GAVI, and UNITAID; instead, these theories may be more applicable and effective when explaining these challenges in older, well established multilateral health agencies, such as the World Bank, WHO, and UNAIDS. Indeed, because the Global Fund was created in 2003, the impact of its preexisting policy ideas and experiences may not be as influential in shaping policy-makers’ decisions when compared to the older World Bank and WHO, multilateral agencies that have been providing donor aid assistance in health for almost half a century. In contrast to the World Bank and the WHO, the Global Fund and GAVI have not been established long enough to create policy legacies and learning processes that generate path dependent *legitimacy* constraints. Future research will need to explore other path dependent and/or other related institutional stasis theories providing insight into the limits to institutional change within recently establish multilateral health agencies.

Nevertheless, limitations also emerge with the author’s usage of institutional change theory. While *conversion* theory, for example, may help to explain policy and organizational transformations within some multilateral health agencies, e.g., the World Bank and UNAIDS, it may not be helpful for explaining other instances of institutional change. This mainly has to do with conversion theory’s dependence on changing external conditions, e.g., international criticisms and pressures, and coalition formation processes between policy-makers within institutions and other external institutional allies as necessary conditions for change to occur. And yet, institutional change may also occur in the absence of these causal conditions. This suggests that *conversion* and *displacement* theories can only explain institutional change when multilateral health agencies confront challenging external environments, such as heightened international pressures and criticisms, and when reformers can find allies in other multilateral agencies that are willing to support their cause.

In the absence of these external conditions, the reform of multilateral health agencies could be aided by alternative institutional change theories. For instance, a sudden change in agency leadership, such as the emergence of a new agency president with bold ideas, interests, and supportive governing board members, may provide the president with the autonomy and political resources needed to pursue institutional and policy change. In this context, one could use theories of institutional *power*[6] in order to show how the combination of an agency leader’s capacity (measured in terms of increased autonomy and resources, i.e., political or financial), as well as the leader’s clear policy vision and commitment, leads to successful institutional change.

For example, future research may wish to explore how the arrival of World Bank President Jim Yong Kim has added greater institutional power to his presidential office (in part, aided by years of US government influence through its appointment of World Bank presidents [50,51,79]) through a combination of heightened governing board support, external political support, and President Kim’s well-establish track record and passion for working on global health issues. With time, given his background and interests we may also see a shift in World Bank priority lending for healthcare projects, a potential move that has concerned other World Bank agency divisions [80].

The limitations of the author’s proposed path dependency and institutional change theories therefore suggest that several different types of institutional theories could be used for conducting the SCA comparative method. The path dependency and institutional change theories used in this article were provided as an example of the potential utility of using institutional theories to guide the selection and comparison of multilateral health agencies. Future research may nevertheless wish to explore the vast array of other institutional theories to see if they can provide stronger insights into understanding the static, evolutionary, and sustainable nature of multilateral health agencies.

## Conclusion

This study has proposed a comparative method for better understanding the transformative and sustainable capacity of multilateral health agencies: i.e., *Sequential Comparative Analysis* (SCA). To the author’s knowledge, SCA is the first attempt to provide a clearly defined, systematic methodological approach to comparing multilateral health agencies. SCA encourages scholars and agency staff to strategically use social science institutional theory, such as path dependency and institutional change theory, in order to select, compare, and better explain the ability of multilateral health agencies to pursue reforms and to sustain them over time. Going forward, this methodological approach suggests that scholars should refrain from selecting and comparing multilateral health agencies based on their ongoing *empirical* challenges. Instead, researchers should let social science institutional theory guide their selection of case studies, comparative analysis, and policy lessons.

## Endnote

^a^YAWS comes from a disease discovered by the WHO in the 1960s. According to Peabody [17], the WHO’s response to YAWS led to practices that the agency adopted for other diseases, such as: 1) holding international symposia; 2) offering fellowships to staff; and 3) prescribing penicillin and more recently, technical meetings, consultative visits and the provision of supplies.

## Abbreviations

AFA: Agenda for Action; ASAP: AIDS Strategic & Action Plan; GAVI: Global Alliance for Vaccines & Immunization; IDA: International Development Association; MAP: Multi-Country AIDS Program; MOPAN: Multilateral Organizational Performance Assessment Network; SCA: Sequential Comparative Analysis; UN: United Nations; UNAIDS: UNESCO: United Nations Educational, Scientific, and Cultural Organization; WHO: World Health Organization.

## Competing interests

The author declares that he has no competing interests.
